# Corona virus disease 2019-associated Stevens-Johnson syndrome: a case report

**DOI:** 10.1186/s12886-021-02033-y

**Published:** 2021-07-12

**Authors:** Toktam Shahraki, Kiana Hassanpour, Amir Arabi, Iman Ansari, Mohammad-Mehdi Sadoughi

**Affiliations:** 1 Ophthalmic Research Center, Research Institute for Ophthalmology and Vision Science, Shahid Beheshti University of Medical Sciences, Tehran, Iran; 2 Department of ophthalmology, Imam Hossein Hospital, Shahid Beheshti University of Medical Sciences, Tehran, Iran; 3 Department of ophthalmology, Labbafinejad Medical Center, Shahid Beheshti University of Medical Sciences, Paidarfard St., Boostan 9 St., Pasdaran, Tehran, 16666 Iran

**Keywords:** COVID-19 - Stevens-Johnson syndrome, Ocular complicatiom- treatment

## Abstract

**Background:**

To report Stevens-Johnson syndrome (SJS) in a patient with acute pneumonia secondary to SARS-CoV-2 infection.

**Case presentation:**

A 45-years-old woman with a diagnosis of acute pneumonia secondary to SARS-CoV-2 infection who had received azithromycin and naproxen. Three days after starting the medication, she appeared ill and developed ocular discomfort, photophobia, dysuria, and macular rashes on the trunk and the extremities. On ophthalmological examination, a total epithelial defect was seen in both eyes. According to the examination, Stevens-Johnson syndrome was diagnosed and the patient was admitted to receive systemic and ocular support and medical care. The patient’s condition improved during the 3 weeks and recovered from both COVID-19 and SJS life-threatening complications but ocular complications, including the destruction of the meibomian glands, irregularity of the eyelid margin, and corneal scarring remained for the patient.

**Conclusions:**

Although, it is not clear whether the cause of Stevens-Johnson syndrome in COVID-19 patients is the virus itself or whether the use of medication, but patients with COVID-19, especially patients receiving medication, should be screened for symptoms of Stevens-Johnson syndrome.

## Background

Severe acute respiratory syndrome coronavirus 2 (SARS-CoV-2) is the viral etiology of the current outbreak of coronavirus disease 2019 (COVID-19) [1]. A wide spectrum of medications, from antiviral agents to immunomodulatory drugs, have been used since the initial days of the pandemic. Besides, traditional anti-inflammatory medications including corticosteroids and non-steroid anti-inflammatory drugs (NSAIDs) have been used to control the severe inflammatory response of the body to SARS-CoV-2.

Stevens-Johnson syndrome (SJS) is a serious disease of the skin and mucous membranes. Special features of host immunity and drug metabolism, in association with special drug structures, consist of the etiology of the disease [2]. From an immunologic standpoint, a delayed-type hypersensitivity reaction to the drug or drug-peptide complexes is the core pathophysiologic event, where cytotoxic T-cells and natural killer cells play the main roles [3]. The medications mostly associated with SJS are anti-convulsion, anti-inflammatory, and antibiotic drugs. Besides, some conditions including viral disease and weakness of the immune system, have been considered as predisposing factors for SJS.

Herein, we report a case of COVID-19 who was treated with naproxen, azithromycin, and corticosteroid. Following a few days from initiation of the treatment, the patient manifested severe mucosal and dermatologic signs, diagnosed as SJS.

## Case presentation

**Fig. 1:**
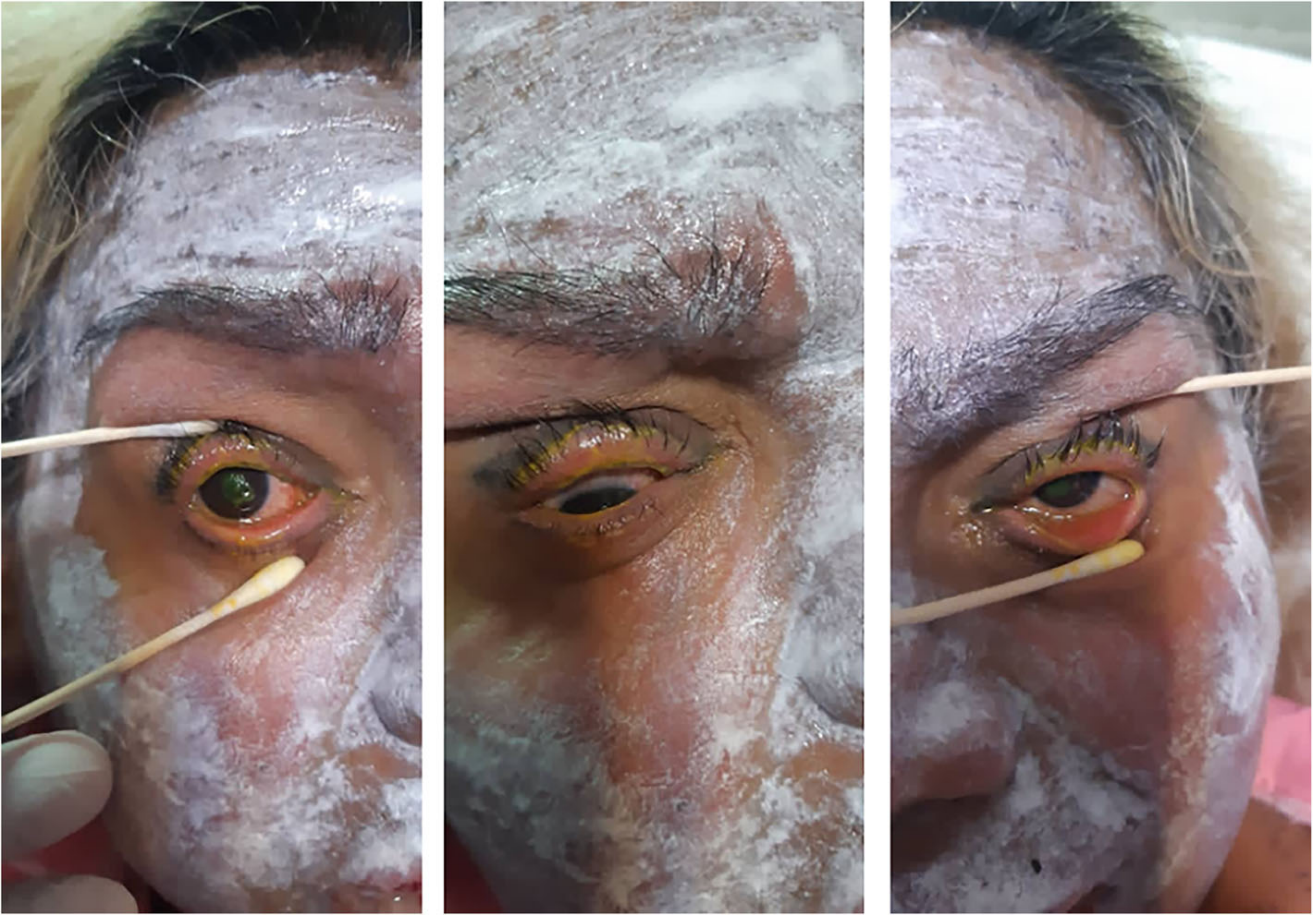
Gross photo of the patient demonstrating skin lesions covered with topical ointments. The right eye (left and middle picture) shows conjunctival hyperemia and discharge. The corneal surface partially shows the epithelial defect. It was impossible to take photo-slit images from the patient at this stage of COVID-19. The left photo shows the left eye with less severe involvement

**Fig. 2:**
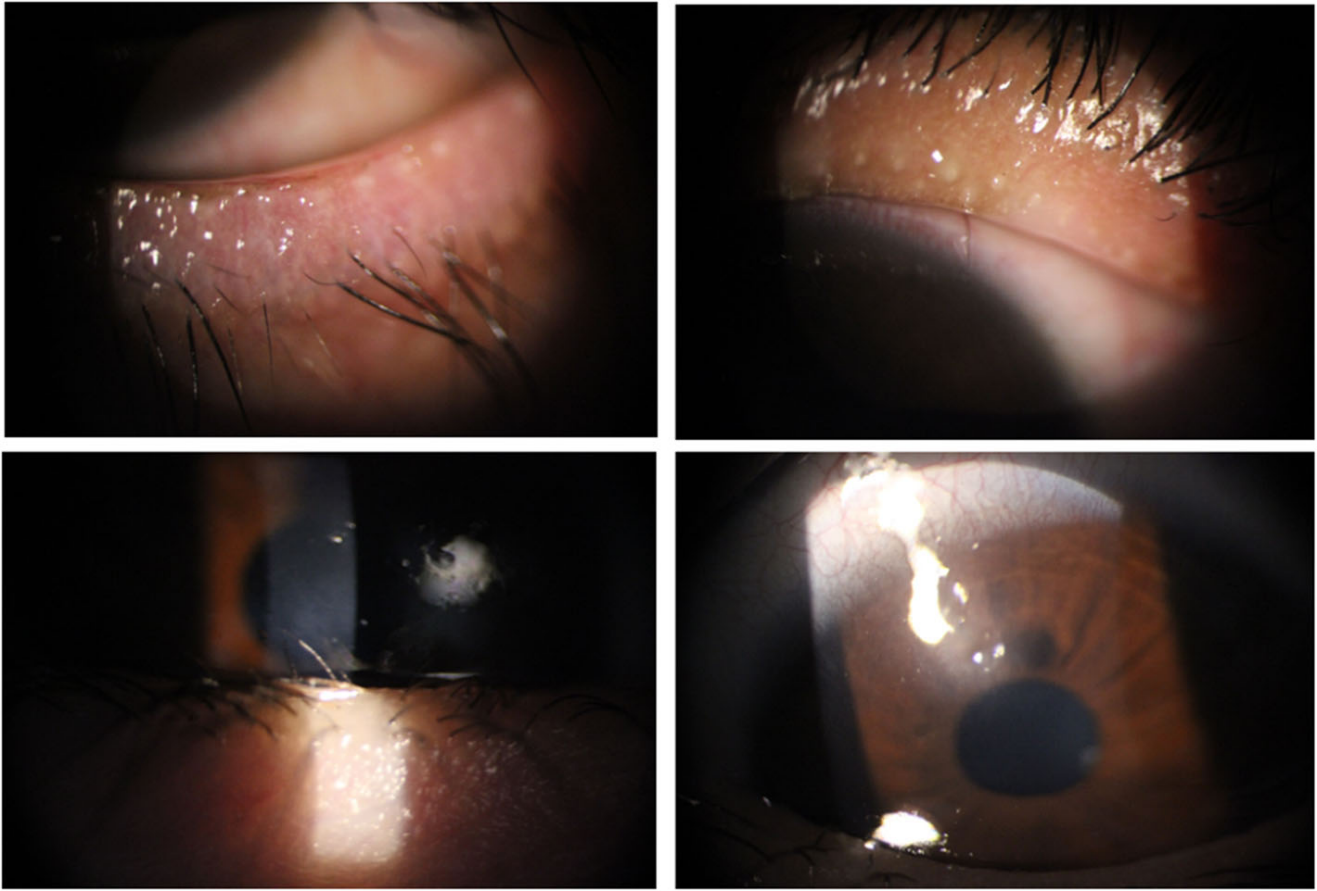
Two upper photo-slit images show disrupted and atrophied Meibomian glands in upper and lower eyelids 3 weeks after the start of the disease. An irregular eyelid margin stained with fluorescein is also observed in the right image. Two lower images demonstrate an area of scar in the stroma of the right eye with overlying epithelial thinning

In April 2020, a 45-year old woman was quarantined with a diagnosis of acute pneumonia secondary to SARS-CoV-2 infection. A nasopharyngeal swab reverse transcription-polymerase chain reaction test had a positive result for COVID-19. Due to mild systemic and respiratory symptoms, azithromycin (Abidipharma Co., Tehran, Iran) (500 mg, bid) and naproxen (Abidipharma Co., Tehran, Iran, 500 mg, bid) was administered to prevent co- or super-infections and control the inflammatory responses. Of note, the patient did not receive any systemic steroid at this stage. On day 3 of treatment, she appeared ill and developed ocular discomfort, photophobia, dysuria, and macular rashes on the trunk and the extremities (Fig. 1). She was admitted to receive oxygen and hydration therapy, and a regimen of intravenous dexamethasone (Aburaihan Pharmaceutical Co., Tehran, Iran, 4 mg, every 8 h) and ceftriaxone (Aburaihan Pharmaceutical Co., Tehran, Iran, 1 g, twice) was started with a diagnosis of SJS. The bedside visual acuity was at least counting fingers from 2 m in the right eye and 4 m in the left eye. Using an indirect ophthalmoscope and 20 lenses, a total epithelial defect was observed in both eyes. During her admission, topical Levofloxacin single dose (Sina Darou Laboratories Co., Tehran, Iran), topical preservative-free Methylpredsnisolone 1% (Prepared by a Compound Pharmacy in Ophthalmic Research Center, Shahid Beheshti University of Medical Sciences, Tehran, Iran), and frequent lubrications were administered. She was visited daily for supportive ocular treatments, including frequent lubrication, full range eye movements, and topical corticosteroid and antibiotics. After 1 week, autologous serum every 6 h was added due to the delayed nature of epithelial defect healing. Her condition improved during the next 3 weeks and recovered from both COVID-19 and SJS life-threatening complications. The patient’s follow-up visits continued on an outpatient basis in the ophthalmology clinic (Fig. 2). The BCVA improved to 20/30 OD and 20/20 OS. A scar was formed in the superotemporal area of the cornea in the right eye. Meibomian glands were severely disrupted and an irregular eyelid margin stained with fluorescein was observed in both eyes.

## Discussion

To investigate the risk factors, both causative medications (naproxen and azithromycin) and primary viral disease may have been the etiologies of SJS in our patient. According to the data presented above, the following scenarios may have predisposed our patient to SJS as a rare medical condition:

1. Although extremely rare, it may have occurred following a short course of naproxen therapy. Long-term usage of NSAIDs (including ibuprofen, naproxen, and celecoxib) are named as causative medications for SJS [4]. The risk of NSAID-associated SJS is extremely low, especially in short-term regimens. Among them, oxicam agents are shown to have the highest association with SJS, while the association for other NSAIDs is much lower.
2. It may have occurred following the administration of azithromycin. Short courses of antibiotics (including sulfonamide and cephalosporin drugs) are associated with SJS. There is also a report of azithromycin-associated SJS, where the patient presented with SJS signs and symptoms after 10 days from taking a course of azithromycin for a respiratory infection [5]. Accordingly, azithromycin may have played the role of the causative agent in our case, which makes our report one of the scarce reports on azithromycin-induced SJS
3. The synergistic effect of simultaneous naproxen and azithromycin is another impression for the etiology of SJS in our case, although this type of synergism never has been reported in the literature.
4. Independent of the medications, primary viral infection may have caused the disease through pathophysiology illustrated previously in HSV and hepatitis-associated SJS reports. The immune system can be activated by virus-associated antigen patterns, as well as viral genomes [5]. To the best of our knowledge, there is no report of SJS directly related to COVID-19.
5. Finally, it should be considered that the coincidence of a rare disease, such as SJS, with a rarely associated drug, such as naproxen and azithromycin, could have been facilitated by the immune stimulation induced by the virus SARSCoV-2. This hypothesis has been reported previously to be the cause of hydroxychloroquine-associated SJS in COVID-19 patients [6].

## Conclusion

Notably, our study is limited by the lack of biopsy confirmation for SJS diagnosis, it seems necessary to monitor the SJS occurrence in COVID-19 positive patients, since it may uncover some facts about the pathophysiology of the SJS, from causative medication contributing to SJS to virus-associated mechanisms involved in the SJS development. We believe that COVID-19 should be considered as an SJS-associated viral infection, especially when well-known SJS-associated drugs (as an additional risk factor) are administered for COVID-19 patients.

## Data Availability

The datasets used and/or analysed during the current study are available from the corresponding author upon request.

## Abbreviations

SJS: Stevens-Johnson syndrome
SARS-CoV-2: Severe acute respiratory syndrome coronavirus 2 COVID-19: coronavirus disease 2019
NSAIDs: Non-steroid anti-inflammatory drugs
BCVA: Best corrected visual acuity
HSV: Herpes simplex virus

## References

[1] Sanders JM, Monogue ML, Jodlowski TZ, Cutrell JB (2020). Pharmacologic treatments for coronavirus disease 2019 (COVID-19): a review. JAMA..

[2] Roujeau JC, Huynh TN, Bracq C, Guillaume JC, Revuz J, Touraine R (1987). Genetic susceptibility to toxic epidermal necrolysis. Arch Dermatol.

[3] Su SC, Chung WH (2014). Cytotoxic proteins and therapeutic targets in severe cutaneous adverse reactions. Toxins..

[4] Keskin Ö, Yıldırım İ, Kalemoglu M, Kucukardali Y, Yüksel F (2005). Naproxen sodium - induced toxic epidermal necrolysis a case report. Nobel Medicus.

[5] Brkljacić N, Gracin S, Prkacin I, Sabljar-Matovinović M, Mrzljak A, Nemet Z (2006). Stevens-Johnson syndrome as an unusual adverse effect of azithromycin. Acta Dermatovenerol Croatica.

[6] Rossi CM, Beretta FN, Traverso G, Mancarella S, Zenoni D (2020). A case report of toxic epidermal necrolysis (TEN) in a patient with COVID-19 treated with hydroxychloroquine: are these two partners in crime?. Clin Mole Allerg.

